# Upregulated microRNA let-7a accelerates apoptosis and inhibits proliferation in uterine junctional zone smooth muscle cells in adenomyosis under conditions of a normal activated hippo-YAP1 axis

**DOI:** 10.1186/s12958-021-00753-w

**Published:** 2021-06-03

**Authors:** Jun-Hua Huang, Hua Duan, Sha Wang, Yi-Yi Wang, Cheng-Xiao LV

**Affiliations:** Department of Minimally Invasive Gynecologic Center, Beijing Obstetrics and Gynecology Hospital, Capital Medical University, 17 Qi Helou Road, Dong Cheng, Beijing, 100006 P.R. China

**Keywords:** Adenomyosis, Let-7a, Apoptosis, Proliferation, Junctional zone, Smooth muscle cells, hippo-YAP1

## Abstract

**Background:**

Let-7a is a small non-coding RNA that has been found to take part in cell proliferation and apoptosis. The hippo-YAP1 axis, known as a tumour suppressor pathway, also plays an important role in cell proliferation and apoptosis. YAP1, TAZ, and phospho-YAP1 play key roles in actions of the hippo-YAP1 axis. Adenomyosis (ADS) is a proliferative disease leading to a large uterus in patients with prolonged illness. Abnormal proliferation of smooth muscle cells (SMCs) in the uterine endometrial-myometrial junctional zone (JZ) is an important reason for developing ADS. This study aimed to explore the expression levels of let-7a and components of the hippo-YAP1 axis in SMCs in the uterine endometrial-myometrial JZ in ADS and to explore the roles of let-7a and the hippo-YAP1 axis of JZ SMC proliferation and apoptosis in ADS.

**Methods:**

We collected JZ tissues for the primary culture of SMCs from 25 women diagnosed with ADS and 27 women without ADS. We used quantitative real-time polymerase chain reaction and western blotting to measure the mRNA and protein expression levels of let-7a, YAP1, TAZ, and phospho-YAP1 in ADS JZ SMCs. A CCK-8 assay and flow cytometry analysis of apoptosis were utilized to test the proliferation and apoptosis of JZ SMCs. The let-7a overexpression lentiviral vector GV280 was used to increase the expression level of let-7a. We added verteporfin to block the phosphorylation of components of the hippo-YAP1 axis.

**Results:**

We found that the let-7a level was decreased, while the YAP1 and TAZ levels were increased in ADS JZ SMCs. Upregulated let-7a affected the expression levels of components of the hippo-YAP1 axis, accelerated apoptosis, and inhibited proliferation in JZ SMCs. Furthermore, accumulated YAP1 led to increasing proliferation of JZ SMCs after verteporfin treatment to block the phosphorylation of components of the hippo-YAP1 axis. If components of the hippo-YAP1 axis were unphosphorylated, upregulated let-7a could not inhibit the proliferation of ADS JZ SMCs. Upregulated let-7a could not activate the hippo-YAP1 axis in verteporfin treatment.

**Conclusions:**

Our findings suggest that the let-7a and hippo-YAP1 axis may act as important regulators of JZ SMCs proliferation, and upregulated let-7a may be an effective method to treat ADS.

## Background

Adenomyosis (ADS) is a common gynecological disease, and its typical characteristic is ectopic endometrium in the myometrium. Abnormal uterine bleeding, progressive dysmenorrhea, and infertility are the major causes that drive patients with ADS to seek treatments [1]. Although this disease has been known for many years, its pathogenesis is still unclear.

The endometrial-myometrial junctional zone (JZ) plays an important role in the development of ADS. Irregular thickening of this zone greater than 12 mm on T2-weighted magnetic resonance imaging has become the key criterion in the diagnosis of ADS [2]. Evidence has shown that in ADS, uterine smooth muscle cells (SMCs) are hypertrophic and have ultrastructural changes [3]. More importantly, uterine peristaltic activity originates from the JZ [4, 5]. Other evidence shows that JZ dysfunction is related to infertility and dysmenorrhea. However, our knowledge of the JZ is still insufficient. It is unclear whether SMCs in the JZ have a differential expression of signaling pathway proteins with intrinsic proprieties to support the development of ADS.

Let-7a is a small non-coding RNA that has been found to take part in cell proliferation and apoptosis in various cancers and other diseases [6, 7]. Modulating the expression of let-7a can inhibit cell proliferation to suppress cancer development [8–10].

ADS is also a proliferative disease, but whether let-7a takes part in the development of ADS is still unknown.

The hippo-YAP1 axis is a tumor suppressor pathway that can control organ size under homeostatic conditions by regulating cell proliferation and apoptosis [11]. YAP1, also known as YAP, represents the major downstream signaling molecule of the hippo-YAP1 axis and is inhibited by the Hippo kinase cascade [12]. When this axis is active, YAP1 and TAZ are phosphorylated and regulate the balance of cell function. When the hippo-YAP1 axis is suppressed, unphosphorylated YAP1 and TAZ enter the cell nucleus and act as transcriptional coactivators of target genes involved in cell proliferation and survival [13].

Most ADS patients have a large uterus, especially those with long-term disease. While the hippo-YAP1 axis can control organ size, whether its dysfunction gives rise to the large uterus observed in ADS remains unclear.

Based on the above evidence, this study explored whether let-7a and the hippo-YAP1 axis affect the onset and progression of ADS.

## Materials and methods

### Sample collection

We collected uterine endometrial-myometrial JZ tissues from 25 women diagnosed with ADS as the experimental group and 27 women without ADS as the control group from January 2020 to July 2020 at the Beijing Obstetrics and Gynaecology Hospital. All patients underwent a hysterectomy. The inclusion criteria in the experimental group included premenopausal women diagnosed with ADS; in the control group included premenopausal women diagnosed with early stage cervical cancer or ovarian cancer without histopathological evidence of ADS. The exclusion criteria included the patients with indication of concomitant fibroid, endometriosis or endometrial pathology or malignancy; and history of intrauterine device placement or hormone therapy within 3 months before surgery. This study was authorized by the ethics committee of our hospital (no. 2016-KY-012). All patients provided signed informed consent before surgery.

### Cell culture

When uteri were obtained within 10 min of surgical removal, we opened in the sagittal plane. After scraping the endometrium to the base layer in the posterior wall of the uterus, multiple 5mm^3^ samples were immediately obtained from the JZ (the first 5–8 mm underneath the endometrium) under optical microscope positioning. We immersed JZ tissues into the saline solution and immediately sent them to the laboratory for subsequent primary cell culture. The JZ tissues were minced into 1-2 mm^3^, digested with 0.2% type I collagenase (Gibco, Carlsbad, CA, USA) containing 0.005% deoxyribonuclease (Invitrogen, Carlsbad, CA, USA) to digest for 4–5 h at room temperature, Afterwards, DMEM/F12 (Hyclone, USA) containing 12.5% fetal bovine serum (FBS; BD, USA) was added and subsequently filtered through the 100 μm cell strainer, centrifuged (1200 x g, 25 °C, 10 min) two times, the final cell suspension was SMCs, cells were incubated in DMEM/F12 supplemented with 12.5% FBS at 37 °C in a 5% CO2 incubator for subsequent experiments.

### Cell treatment

The let-7a overexpression lentiviral vector GV280: hU6-MCS-Ubiquitin-EGFP-IRES-puromycin (Shanghai GeneChem Co., Ltd., Shanghai, China) was transfected at a multiplicity of infection of 10 (1 × 10^7^ TU/ml) for 12 h at 37 °C after cells were grown to a density of 30–40%. After 72 h, the transfection efficiency was observed using white-light microscopy and fluorescence microscopy. Subsequently, the cells were selected using 1 μg/ml puromycin (Beijing Solarbio Science & Technology Co., Ltd., Beijing, China) until there were no dead cells in the culture plates. Transfected cells were used for western blot, RT-qPCR, and cell function assays.

Verteporfin (Beijing Solarbio Science & Technology Co., Ltd., Beijing, China) was used at a dose of 1 μM for 24 h at 37 °C to treat SMCs to block the hippo-YAP1 axis. Verteporfin was dissolved in dimethyl sulfoxide (DMSO; Sigma, Missouri, USA). We used DMSO as the control treatment at a dose of 1 μM for 24 h at 37 °C to treat smooth muscle cells and then harvested the cells for subsequent experiments.

### RNA extraction and quantitative real-time polymerase chain reaction (qRT-PCR)

For let-7a analysis, total RNA was extracted according to the manufacturer’s instructions for RNAiso Plus (Takara Bio Inc., Japan). Then, the total RNA was converted to cDNA using a Mir-X miRNA First-Strand Synthesis kit (Takara Bio, Inc., Japan). We used a 2-step quantitative polymerase chain reaction (PCR) approach according to the protocol of TB Green Advantage qPCR Premix (Takara Bio, Inc., Japan) on an ABI 7500 Real-Time PCR system (Applied Biosystems; Thermo Fisher Scientific, Inc., USA). For YAP1 and TAZ analysis, TRIzol reagent (Invitrogen, Carlsbad, CA, USA) was used to extract total RNA, and a NanoDrop 2000 spectrophotometer (Thermo Fisher Scientific, Inc., USA) was used to confirm RNA quality. Subsequently, we used a FastQuant RT kit (Tiangen Biotech Co., Ltd., China) to synthesize cDNA and evaluated the template cDNA by qPCR with the SuperReal PreMix Plus kit (Tiangen Biotech Co., Ltd., China) according to the manufacturer’s protocol. The comparative CT method [14] was used to analyze relative gene expression levels. The primers sequences are shown in Table 1.

**Table 1:**
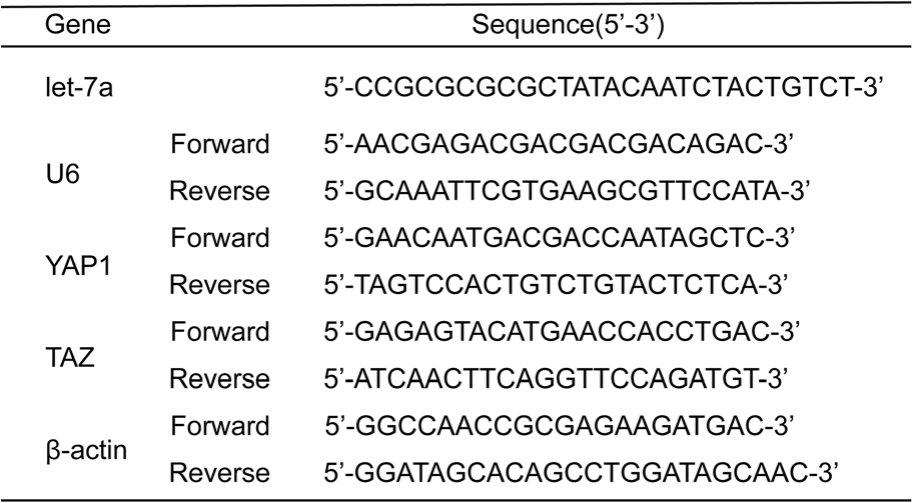
Primers of specific genes used in qRT-PCR analyses

### Western blotting

RIPA buffer (Beijing Solarbio Science & Technology Co., Ltd., Beijing, China) was used to acquire total protein, and the BCA Protein Assay Kit (Beyotime Institute of Biotechnology, China) was used to test the protein concentration. Sodium dodecyl sulfate-polyacrylamide gels (6, 12, and 15%, Beijing Solarbio Science & Technology Co., Ltd., Beijing, China) were used to separate the protein, which was then transferred to polyvinylidene fluoride membranes (EMD Millipore). The membranes were blocked for 2 h with 5% milk at room temperature. Next, the membranes were washed and incubated with an anti-YAP1 rabbit monoclonal antibody (Beyotime Institute of Biotechnology, China), an anti-phospho-YAP1 rabbit monoclonal antibody (1:1000; Beyotime Institute of Biotechnology, China), an anti-TAZ rabbit polyclonal antibody (1:1000, Beyotime Institute of Biotechnology, China), and an anti-α-tubulin rabbit monoclonal antibody (1:1000, Cell Signalling Technology) overnight at 4 °C with gentle agitation. After that, we washed the membranes with Tris-buffered saline-Tween 20 (Beijing Solarbio Science & Technology Co., Ltd., China) 3 times and incubated them with HRP-linked anti-rabbit IgG (1:2000, Cell Signalling Technology) and HRP-linked anti-mouse IgG (1:2000, Cell Signalling Technology) for 1 h with gentle agitation at room temperature. Immunoreactive bands were detected using Chemiluminescent HRP Substrate (EMD Millipore). ChemiDoc TM XRS+ and Image Lab software 3.0 (Bio-Rad Laboratories, Inc.) were used to acquire results.

### Cell counting kit (CCK)-8 assays

We used a CCK-8 (Dojindo Molecular Technologies, Inc., Japan) assay to assess cell proliferation. We cultured JZ SMCs at 4000 cells/well in 96-well plates at 37 °C in 5% CO2. We added a CCK-8 solution (10 μl) to each well at different time points (12, 24, 36, 48, 60, 72, 84, and 96 h) and incubated the plates for 4 h. Finally, a microplate reader was used to test the absorbance at 450 nm.

### Flow cytometry analysis of apoptosis

An Annexin-V-APC/PI double-staining kit (KeyGen Biotech Co., Ltd., China) was used to detect apoptotic cells according to the manufacturer’s protocol. After treated cells were detached from culture plates with 0.25% trypsin (without EDTA) (Gibco, Carlsbad, CA, USA), we washed them twice with phosphate-buffered saline (PBS) and centrifugation (2000 x g, 25 °C, 5 min). Then, the cells were resuspended in binding buffer and stained with both annexin V-APC and PI for 15 min in the dark. Finally, we used flow cytometry (BD FACSCalibur™ cytometer, BD Biosciences) to detect stained cells.

### Statistical analysis

All data were analyzed by GraphPad Prism 8.4 (GraphPad Software, Inc., La Jolla, CA, USA). Data are represented as the mean ± standard deviation (SD). We used an unpaired Student’s t-test or the Wilcoxon signed-rank test to compare differences between the two groups. One-way ANOVA or the Kruskal-Wallis H test was used to compare three groups. For all tests, a *P* value < 0.05 was considered significant.

## Results

### Let-7a is downregulated, while YAP1 and TAZ are upregulated in ADS

**Fig. 1:**
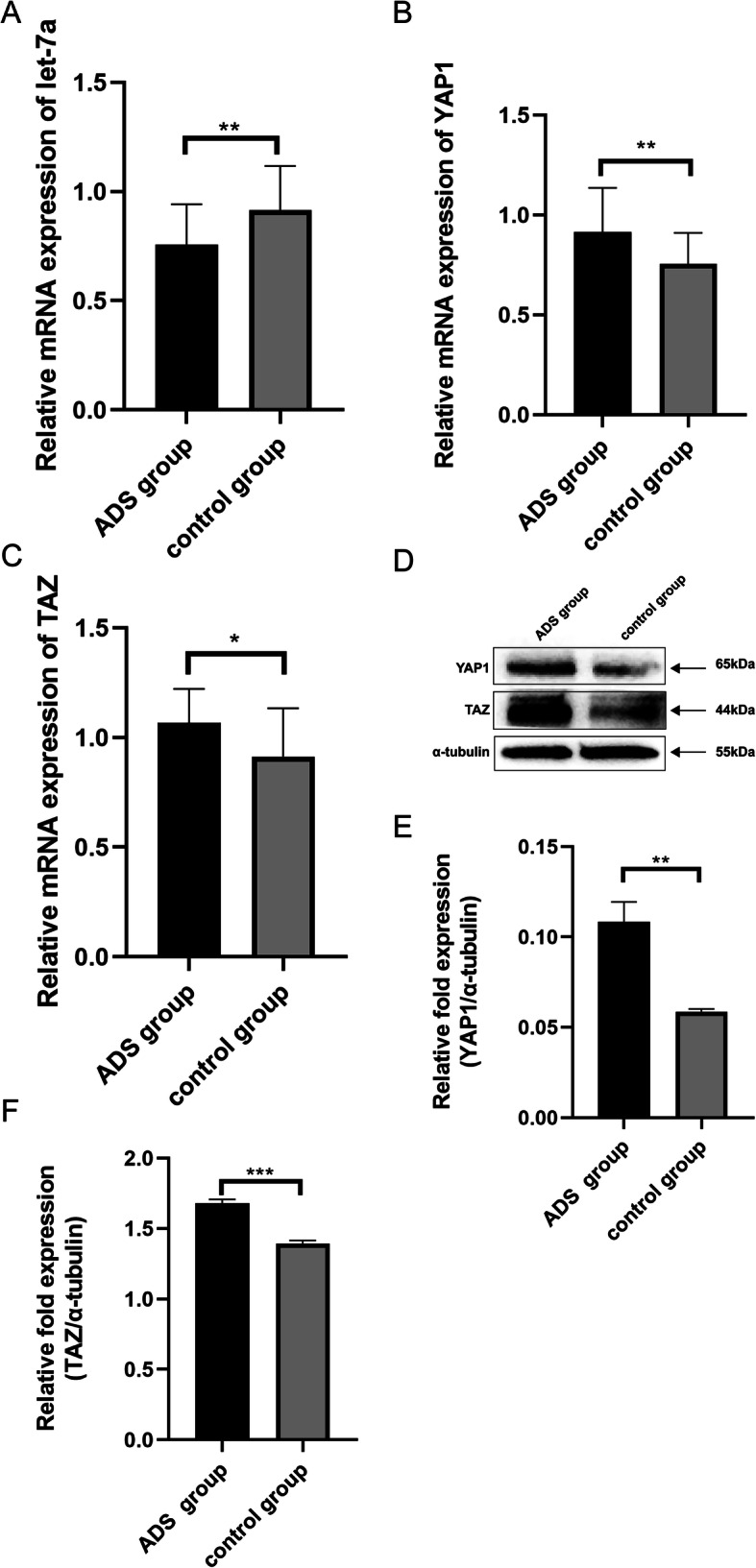
The expression levels of let-7a, YAP1 and TAZ in JZ SMCs in the ADS group and control group. **a** The mRNA expression level of let-7a determined by RT-qPCR and normalized against that of U6. **b** The mRNA expression level of YAP1 determined by RT-qPCR and normalized against that of β-actin. **c** The mRNA expression level of TAZ determined by RT-qPCR and normalized against that of β-actin. **d** The protein expression levels of YAP1 and TAZ determined by western blotting and normalized against that of α-tubulin. **e** The relative fold protein expression of YAP1 normalized against α-tubulin. **f** The relative fold protein expression of TAZ normalized against α-tubulin. These experiments were performed two times with three replicates in each experiment. Significance was determined by Student’s t test; **p < 0.01, *p < 0.05

To determine whether there is differential expression of let-7a, YAP1, and TAZ in JZ SMCs, we used qRT-PCR and western blotting to test their basal expression in an ADS group and a control group. Interestingly, the mRNA expression of let-7a was lower in the ADS group than in the control group (Fig. 1a). However, the mRNA expression of YAP1 (Fig. 1b) and TAZ (Fig. 1c) in the ADS group was higher. Then, we further tested the protein expression of YAP1 (Fig. 1e) and TAZ (Fig. 1f), and the results were consistent with the qRT-PCR results. From these results, we suspected that let-7a might affect the hippo-YAP1 axis in ADS to promote ADS development.

### Upregulated let-7a affects the expression level of YAP1 and TAZ

**Fig. 2:**
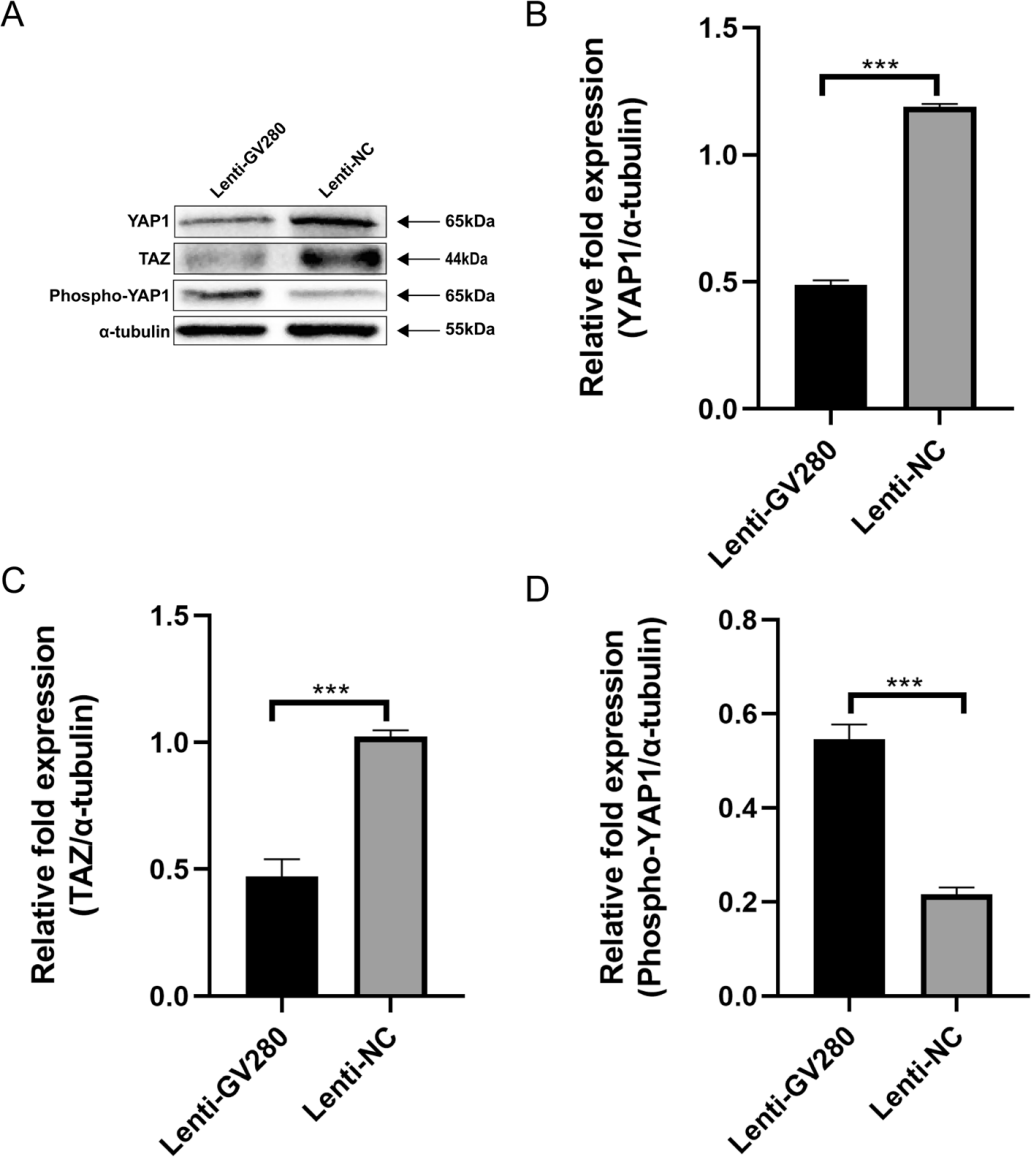
The expression levels of YAP1, TAZ and phospho-YAP1 in JZ SMCs in the ADS group. **a** Cells were transfected with the let-7a overexpression lentiviral vector GV280 or lentiviral null vector for 72 h and then used for western blotting. **b** The relative fold protein expression of YAP1 normalized against α-tubulin. **c** The relative fold protein expression of TAZ normalized against α-tubulin. **d** The relative fold protein expression of phospho-YAP1 normalized against α-tubulin. These experiments were performed two times with three replicates in each experiment. Significance was determined by Student’s t test; ****p < 0.0001, ***p < 0.001

We upregulated let-7a in ADS SMCs with the let-7a overexpression lentiviral vector GV280 (Lenti-GV280). Then, through western blotting, we found that the expression of YAP1 was obviously lower in the Lenti-GV280 group than in the control group (Fig. 2a and Fig. 2b); moreover, TAZ was also downregulated in the Lenti-GV280 group (Fig. 2a and Fig. 2c). However, the expression of phospho-YAP1 was higher (Fig. 2a and Fig. 2d). According to these results, we concluded that let-7a actually affects the expression of YAP1 and TAZ in the hippo-YAP1 axis. Furthermore, let-7a may participate in activating the hippo-YAP1 axis via upregulation of phospho-YAP1.

### Upregulated let-7a inhibits proliferation and accelerates apoptosis in JZ SMCs in ADS

We transfected the let-7a overexpression lentiviral vector GV280 or lentiviral null vector into JZ SMCs, and then a CCK-8 assay and flow cytometry analysis were used to test the proliferation and apoptosis of the ADS JZ SMCs. The results showed that upregulated let-7a significantly increased early and late apoptosis in ADS JZ SMCs compared with Lenti-NC treatment (Fig. 4a and Fig. 4b). Furthermore, the 450-nm absorbance of the Lenti-GV280 group was lower than that of the Lenti-NC group (Fig. 4e).

### Verteporfin inhibits the phosphorylation of YAP1, leading to an elevated proliferation of JZ SMCs

**Fig. 3:**
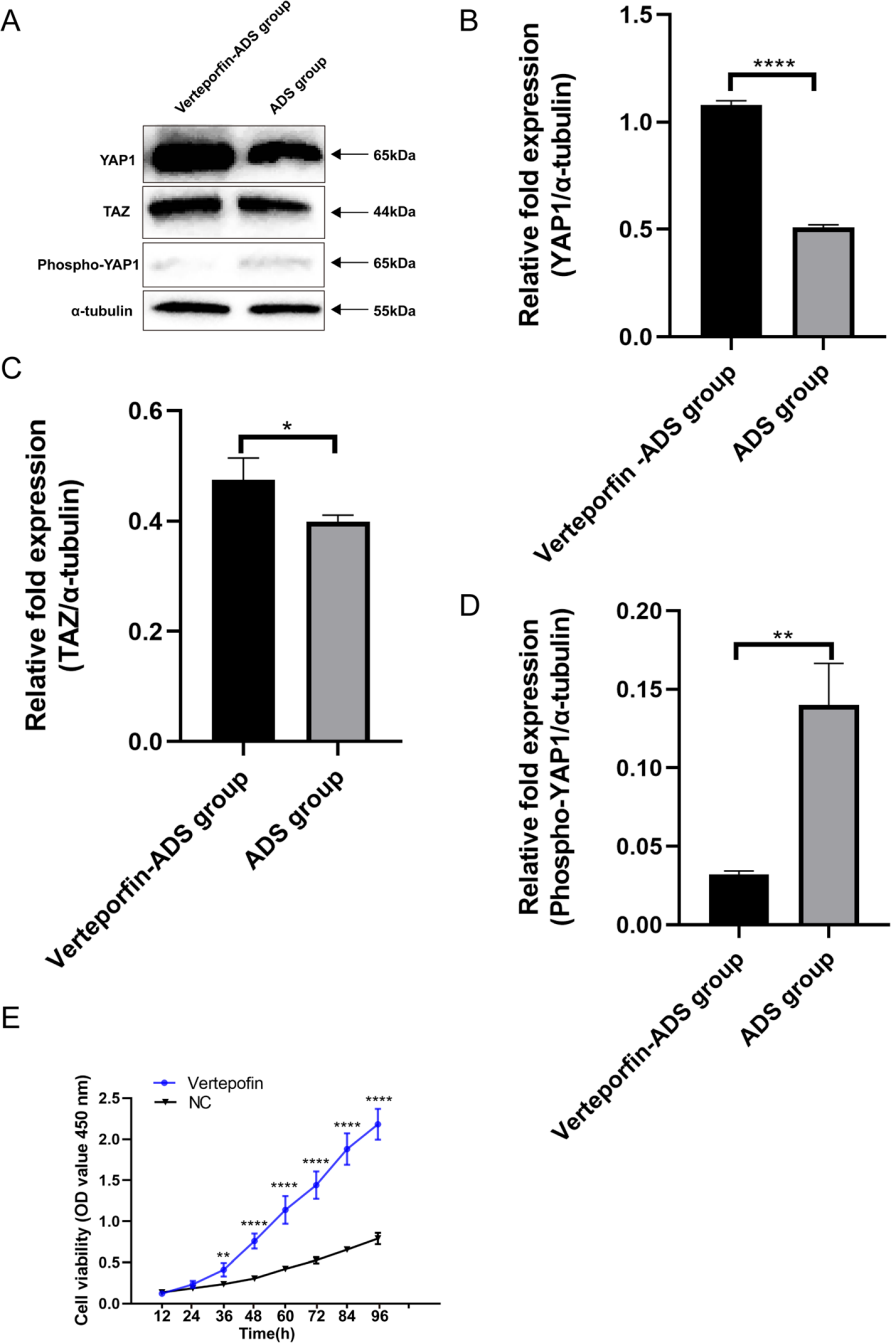
The expression levels of YAP, TAZ and phospho-YAP1 in JZ SMCs in the verteporfin-ADS group and ADS group and CCK-8 assay results for the verteporfin-ADS group and negative control group. **a** Cells were co-cultured with 1 μM verteporfin and then used for western blotting. **b** The relative fold protein expression of YAP1 normalized against α-tubulin. **c** The relative fold protein expression of TAZ normalized against α-tubulin. **d** The relative fold protein expression of phospho-YAP1 normalized against α-tubulin. **e** Cells were treated with verteporfin, and then a CCK-8 assay was used to test the proliferative ability of ADS JZ SMCs. These experiments were performed two times with three replicates in each experiment. Significance was determined by Student’s t test; ****p < 0.0001, **p < 0.01, *p < 0.05

Because phosphorylated YAP1 plays a key role in the Hippo-YAP kinase cascade axis and we found that YAP1 is upregulated in JZ SMCs, we next used verteporfin to block the phosphorylation of YAP1 and detected the role of YAP1 in SMCs. After SMCs were treated with verteporfin, we used western blotting to test the blocking effect, and the results showed that the expression of TAZ was higher in the verteporfin-ADS group than in the control group (Fig. 3a and Fig. 3c). The expression of YAP1 was significantly elevated in the verteporfin-ADS group (Fig. 3a and Fig. 3b). However, there was little expression of phospho-YAP1 in the verteporfin-ADS group (Fig. 3a and Fig. 3d). A CCK-8 assay showed that verteporfin-treated cells had a higher OD value after 36 h. If YAP1 was not phosphorylated and accumulated in the cytoplasm, it would increase the cell proliferation of JZ SMCs (Fig. 3e).

### Upregulated let-7a accelerates apoptosis and inhibits proliferation in JZ SMCs in the context of an activated hippo-YAP1 axis in ADS

**Fig. 4:**
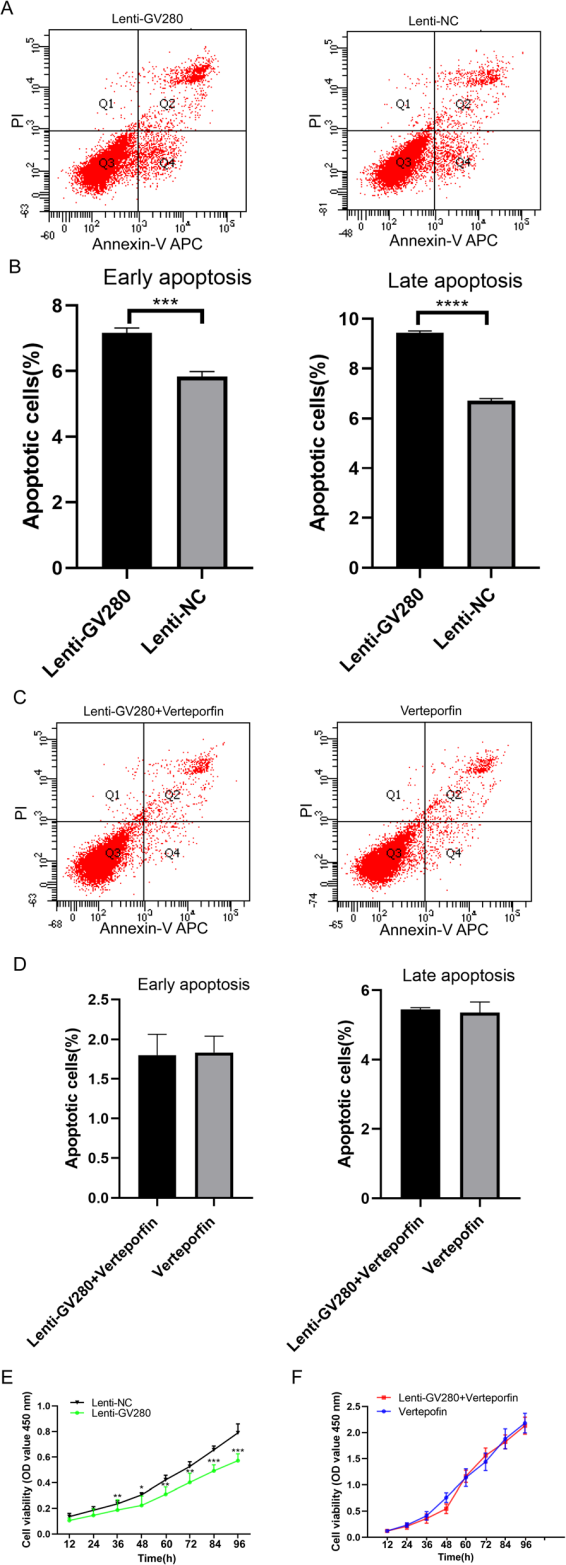
Flow cytometry analysis of apoptosis and a CCK-8 assay performed with ADS JZ SMCs. **a** Cells were transfected with the let-7a overexpression lentiviral vector GV280 or lentiviral null vector for 72 h and then used for flow cytometry analysis. **b** The percentages of apoptotic cells in the lenti-GV280 group and lenti-NC group. **c** Verteporfin-treated cells were transfected with the let-7a overexpression lentiviral vector GV280 for 72 h and then used for flow cytometry analysis. **d** The percentages of apoptotic cells in the lenti-GV280 + verteporfin group and verteporfin group. **e** OD_450_ values of the lenti-GV280 group and lenti-NC group. **f** OD_450_ values of the lenti-GV280 + verteporfin group and verteporfin group. These experiments were performed two times with three replicates in each experiment. Significance was determined by Student’s t test; ****p < 0.0001, ***p < 0.001, **p < 0.01, *p < 0.05

Since the Hippo-YAP axis is known as a tumor suppressor pathway, let-7a also inhibits cell proliferation, and let-7a affects the expression of YAP1 and TAZ. We hypothesized that let-7a may inhibit SMC proliferation by activating the hippo-YAP1 axis. We first added verteporfin to block the hippo-YAP1 axis and then transfected the let-7a overexpression lentiviral vector GV280 into JZ SMCs to determine whether let-7a can activate the hippo-YAP1 axis to synergistically inhibit SMC proliferation. A CCK-8 assay and flow cytometry analysis were used to test the proliferation and apoptosis of JZ SMCs in ADS. To our surprise, the lenti-GV280 + verteporfin group had a similar apoptosis rate to the verteporfin group (Fig. 4c and Fig. 4d). The 450 nm absorbance of SMCs in the lenti-GV280 + verteporfin group was also similar to that of those in the verteporfin group (Fig. 4f). These results showed that if the hippo-YAP1 axis was inactivated, let-7a could not promote apoptosis in ADS JZ SMCs. Let-7a could not activate the verteporfin-treated hippo-YAP1 axis. Let-7a exerted an effect in the context of an activated hippo-YAP1 axis.

## Discussion

Let-7a is a microRNA that has necessary functions in cell survival, proliferation, and apoptosis. Many studies have found that let-7a is abnormally expressed in many cancers. Wu et al. [15] indicated that the expression of let-7a was reduced in cervical cancer patients. Overexpression of let-7a also inhibited HeLa cell proliferation. Low expression of let-7a is associated with growth and invasion in oral squamous cell carcinoma [16]. Recurrent prostate cancer has decreased expression of let-7a [17]. Previous studies have explored the role of let-7a in many kinds of cancers, but there have been few studies on other diseases, such as ADS. Although ADS is not a type of cancer, it has abnormal proliferative characteristics that ultimately lead to a large uterus. Our present study verified that let-7a was downregulated in JZ SMCs in ADS. We further increased the expression of let-7a to examine its functions in cell proliferation and apoptosis. The results indicated that upregulated let-7a inhibited cell proliferation and sped up cell apoptosis. Through our study, we found that let-7a played a role not only in cancers but also in ADS. Perhaps let-7a reacts as long as there is abnormal proliferation to maintain the stability of the cell dynamics of the body, so we cannot ignore it.

The hippo-YAP1 axis is a kinase cascade pathway that includes a series of regulatory and scaffolding proteins that take part in many cell functions. YAP1 and TAZ are the main downstream phosphorylation target proteins in the hippo-YAP1 axis. The localization and phosphorylation of YAP1 often reflect the activity of the hippo-YAP1 axis. When YAP1 and TAZ are phosphorylated, they cannot enter the nucleus to bind to target genes and induce proliferation. When YAP1 and TAZ are unphosphorylated, in other words, the hippo-YAP1 axis is inactivated, YAP1 and TAZ can enter the nucleus and act as transcriptional coactivators, leading to cell proliferation. Previous studies have indicated that the YAP1 protein is elevated in various human cancers and that dysregulation of YAP1 may contribute to tumorigenesis [18, 19]. Although cancers are a great threat to human life and health, we cannot neglect the suffering brought by other diseases, such as ADS causing infertility and aggravating pain in women of childbearing age. From this point, our study detected the expression of YAP1 and TAZ in ADS JZ SMCs and found that YAP1 and TAZ were both upregulated in JZ SMCs, and this result might validate the proliferative roles of YAP1 and TAZ in ADS. Then, we used verteporfin to inhibit the phosphorylation of YAP1 and examined the proliferative ability of JZ SMCs. Verteporfin-treated cells had a higher proliferative ability than control cells. This result was consistent with findings in previous studies and verified that accumulated unphosphorylated YAP1 could increase JZ SMC proliferation. Making sure that downstream components of the hippo-YAP1 axis are regularly phosphorylated may be beneficial for the control of ADS development.

Previous studies have shown that microRNAs regulate the core components of the hippo-YAP1 axis, and these relationships cannot be ignored. Modulation of microRNA-125a regulates cancer stem cells through TAZ [20]. MicroRNA-135b binds to LATS2, a member of the hippo-YAP1 axis, inducing cell proliferation, migration, and invasion in breast cancer [21]. Overexpressed microRNA-186 inhibits cell proliferation and migration by downregulating YAP1 in hepatocellular carcinoma [22]. MicroRNA-9 and microRNA-137 regulate the hippo-YAP1 axis, giving rise to the development of gastric cancer [23]. From this point of view, we investigated the relationship between let-7a and the axis. As let-7a and the hippo-YAP1 axis are both related to cell proliferation and apoptosis, we further explored whether let-7a mediates the phosphorylation of components of the hippo-YAP1 axis to synergistically participate in the development of ADS. When we upregulated let-7a in verteporfin-treated SMCs, we found unexpected results. Let-7a did not affect verteporfin-treated SMCs, and there were no significant changes between the lenti-GV280 + verteporfin group and the verteporfin group regarding SMC proliferation and apoptosis. These results showed that only under the condition of a normal activated hippo-YAP1 axis could make upregulated let-7a accelerate SMC apoptosis and inhibit proliferation. We confirmed the necessary role of the normal activated hippo-YAP1 axis in ADS control.

The endometrium is an essential component of the female uterus. The role of non-coding RNAs in endometrial physiology as well as in other endometrial pathologies such as endometriosis and chronic endometritis have also been reported. MicroRNA-27a-3p and microRNA-124-3p were upregulated in the endometrium of chronic endometritis and they might represent non-invasive markers of chronic endometritis, which had a potential value to assess the endometrial quality in IVF [24]. Overexpressed long non-coding RNA (LncRNA) LINC00261 inhibited cell proliferation and migration, promoted cell apoptosis in endometriosis [25]. LncRNA HOTAIR promoted endometrial fibrosis by activating the TGF-β1/ Smad pathway [26]. Endometrial receptivity and implantation were also related to non-coding RNAs [27]. Not only the dysregulation of non-coding RNAs led to an abnormal endometrium but also dysregulation of some apoptotic pathways [28] such as Fas/FasL system [29] and tumor necrosis factor (TNF) -α and tumor necrosis factor receptor (TNFR) 1/tumor necrosis factor receptor (TNFR) 2 system [30] gave rise to endometrial diseases. Long-term endometrial lesions increased the risk of endometrial cancer (EC). ADS and EC share several altered molecular pathways, such as high level of vascular endothelial growth factor, platelet-derived growth factor, increased production of reactive species of oxygen and pro-inflammatory cytokines, KRAS mutations [31], which lead to increased angiogenesis, abnormal tissue growth, and invasion [32]. Besides, pre- or perimenopausal women with ADS can cause abnormal uterine bleeding, which can mislead in the diagnosis of EC [33]. Therefore, early detection and treatment of endometrial diseases such as ADS become extremely urgent. Our study provided a new and effective theoretical basis for the treatment of ADS from the perspective of let-7a and hippo-YAP1 axis.

This study is a preliminary exploration of the relationship between let-7a and the hippo-YAP1 axis in ADS JZ SMCs. We found the deregulation of let-7a not only affected cancer cell proliferation but also led to common gynecological diseases such as ADS. This finding could be a supplement to the function of the let-7a in other diseases besides cancers. Our study might provide a new effective theoretical basis for the treatment of ADS. But there are some limitations in our study. First, we did not test the expression and functions of the let-7a and hippo-YAP1 axis in adenomyotic cells (ectopic endometrial cells within the myometrium) and in myometrial cells around adenomyotic foci to support our hypothesis. Second, given the moderate sample size, some differences could be masked, a larger sample size might be more convincing. Next, we will explore these pathways in adenomyotic cells (ectopic endometrial cells within the myometrium) or myometrial cells around adenomyotic foci, expand the sample size, and establish an animal model to determine whether the hippo-YAP1 axis participates in uterine size changes and identify the function of let-7a in this process. Furthermore, we will collect clinical data to analyze how the hippo-YAP1 axis affects the development of a large uterus in long-term ADS patients.

## Conclusions

In conclusion, we found that let-7a was decreased, while YAP1 and TAZ were increased in ADS JZ SMCs. Furthermore, verteporfin blocked the phosphorylation of YAP1, inducing increased proliferation of JZ SMCs. Let-7a affected the expression level of components of the hippo-YAP1 axis, and upregulated let-7a could accelerate apoptosis and inhibited proliferation in JZ SMCs under conditions of an activated hippo-YAP1 axis in ADS.

## Data Availability

All data generated or analyzed during this study are included in this published article.
